# Autorefraction, Retinoscopy, Javal's Rule, and Grosvenor's Modified Javal's Rule: The Best Predictor of Refractive Astigmatism

**DOI:** 10.1155/2016/3584137

**Published:** 2016-10-10

**Authors:** Kofi Asiedu, Samuel Kyei, Emmanuel Ekow Ampiah

**Affiliations:** ^1^Refraction and Low Vision Clinic, Komfo Anokye Teaching Hospital Eye Center, P.O. Box 1934, Kumasi, Ghana; ^2^Department of Optometry, School of Allied Health Sciences, College of Health and Allied Science, University of Cape Coast, Cape Coast, Ghana; ^3^Ophthalmology Unit, Cape Coast Teaching Hospital, Cape Coast, Ghana

## Abstract

The aim of the study was to determine the level of agreement between Javal's rule, autorefraction, retinoscopy, and refractive astigmatism and to determine which technique is the most suitable substitute when subjective refraction is not applicable using a clinical sample. A total of 36 subjects, 14 males and 22 females, were involved in this study. The intraclass correlation coefficients between subjective refraction, autorefraction, and retinoscopy were 0.895 and 0.989, respectively, for the spherical equivalent. The Bland-Altman 95% limits of agreement between subjective refraction and autorefraction; subjective refraction and retinoscopy; and autorefraction and retinoscopy were −2.84 to 3.58, −0.88 to 1.12, and −3.01 to 3.53, respectively, for the spherical equivalent. The intraclass correlation coefficients between spectacle total astigmatism and the following techniques were as follows: retinoscopy (0.85); autorefraction (0.92); Javal's rule (0.82); and Grosvenor et al. version (0.85). The Bland-Altman 95% limits of agreement between subjective refraction and autorefraction; subjective refraction and retinoscopy; subjective refraction and Javal's rule; and subjective refraction and Grosvenor et al. version were −0.87 to 1.25, −1.49 to 1.99, −0.73 to 1.93, and −0.89 to 1.7, respectively, for the total astigmatism. The study showed that autorefraction and Javal's rule may provide a starting point for subjective refraction cylinder power determination but only retinoscopy may satisfactorily replace subjective refraction total astigmatism when subjective refraction is not applicable.

## 1. Introduction

Objective refraction remains an integral part of the refraction process, as it allows a nonsubjective way of estimating the magnitude of refractive errors. Over the years, there is evidence and consensus that objective refraction still does not replace subjective refraction [1–8]. There are situations wherein subjective refraction is not applicable such as in young children and toddlers [8] and patients with neurological deficits whose subjective responses are not reliable.

There are also situations where the ocular media have obstructions such as cataracts, vitreous hemorrhage, and hyphema which prevent adequate objective refraction results. In such cases, obtaining adequate total astigmatism is problematic. There may be many of such instances where the cornea may be normal and for that reason keratometry readings may be helpful in estimating the total astigmatism. Javal's rule attempts this possibility of estimating total astigmatism from keratometry reading [9, 10]. However, this rule has been modified by Grosvenor et al. [10] based on evidence from research into a simplified version [10]. Several versions of Javal's rule also exist including Sutcliff's rule and O'Shea's rule [10]. A common weakness of these modified versions is their relative complexity lending them to less usage in clinical practice [10].

The proponents for the use of Javal's rule or the simplified version by Grosvenor et al. [10] highlight its benefits in estimating total astigmatism in patients with problems with communication and senile lens changes wherein retinoscopy and autorefraction are unreliable [9, 10]. For instance, some researchers studied the lens as a potential source of astigmatism but found that it was not [9]. However, others found a very small correlation between lenticular astigmatism and cataracts [9].

Elliott et al. [9] reported that Javal's rule is of limited clinical value compared to autorefraction and retinoscopy in estimating total or refractive spectacle astigmatism [4]. However, they came to this conclusion after comparing Javal's original rule and its Grosvenor et al. [10] version in estimating spectacle astigmatism with subjective refraction refined by the Jackson cross cylinder in their study with findings of other studies which found much more accurate prediction of spectacle astigmatism with retinoscopy and autorefraction [9, 11, 12]. One could undoubtedly argue that a different conclusion could have been reached if autorefraction and retinoscopy were done in the Elliott et al. [9] study on the same subjects rather than comparing their findings with those of other studies.

In this study, autorefraction and retinoscopy are compared with each other and their agreement with subjective refraction spherical equivalent and total astigmatism. Also Javal's original rule, Grosvenor et al. version, retinoscopy, and autorefraction estimation of total astigmatism were compared with subjective refraction to determine which method was the best in estimating spectacle total astigmatism. Furthermore, autorefraction and retinoscopy prediction of the cylinder axis were compared with subjective refraction to determine which method is the best in predicting the axis of spectacle astigmatism.

## 2. Methods

This was a hospital based cross-sectional study. Participants were purposely sampled. All participants were patients visiting the Komfo Anokye Teaching Hospital Eye Center. Participants were examined at the eye center's general clinic by a specialist ophthalmologist or consultant ophthalmologist after which they were referred to the refraction clinic. Participants were included in the study if they had no significant ocular pathology. All subjects had healthy corneas. Informed consent was sought from the participants and each participant underwent thorough refractive assessment.

In order to avoid bias, noncycloplegic retinoscopy was performed by an optometrist who was masked to the results of the autorefraction and autokeratometry findings. Noncycloplegic autorefraction and autokeratometry were performed using the same Humphrey Zeiss 599 autorefractor/keratometer. Humphrey Zeiss 599 autorefractor/keratometer is a reliable and accurate instrument which is commercially available, often utilized in clinical practice and in several clinical studies [13, 14]. All subjects underwent subjective refraction with refinement of the cylinder power and axis with a ±0.25 Jackson cross cylinder. Participants who were finally included in the study had their two meridians within 15° of 90 and 180.

After autokeratometry reading, Javal's original rule (1.2 (keratometric astigmatism) −0.50 × 90) and Grosvenor et al. modified Javal's rule (keratometric astigmatism −0.50 × 90) were used to determine refractive or total astigmatism. Ethical clearance for this study was obtained from the Department of Optometry Ethics Review Committee. All procedures and protocol for the study were conducted in accordance with the tenets of the declaration of Helsinki.

### 2.1. Statistical Analysis

All statistical analyses were performed using SPSS V.21.0 (SPSS, Chicago, IL, USA) statistical package and *p* < 0.05 was deemed statistically significant. Intraclass correlation coefficients (ICCs) of the various parameters between the right and left eyes were determined, utilizing the recommendation by Armstrong [15] which states “if both eyes are included in a study, the correlation between both eyes should be assessed using the ICC. If the correlation is close to one, then the data from both eyes could be averaged or one eye selected at random for the analysis using conventional statistics.”

Hence, in this study if the intraclass correlation between the left and right eyes was ≥0.75 then the right eye was arbitrarily selected for the analysis. The agreement between techniques or methods was determined using the intraclass correlation coefficient and Bland-Altman analysis. If there existed proportional bias, the natural logarithm of the values of the techniques was used in the Bland-Altman analysis. All comparisons between techniques with *p* > 0.05 for the mean difference are represented graphically by the Bland-Altman plots. Also the kappa statistics were used to determine autorefraction and retinoscopy prediction of the axis of the spectacle astigmatism as determined by subjective refraction.

## 3. Results

**Figure 1:**
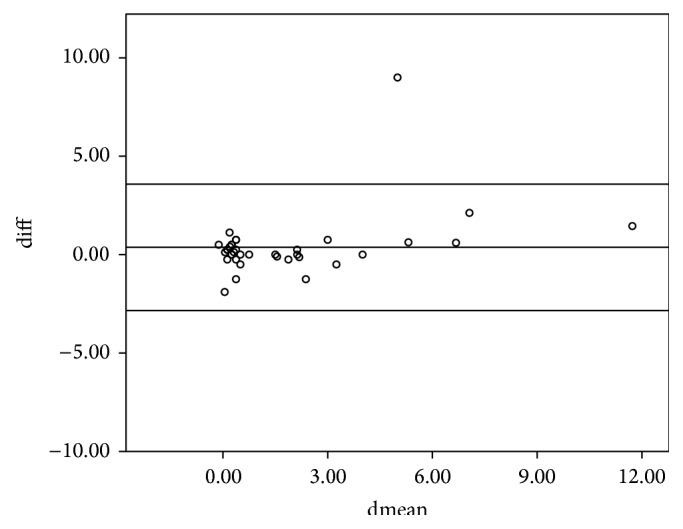
Bland-Altman plots comparing refractive measurements between autorefraction and retinoscopy (spherical equivalent). Difference between the measurements (diff) is plotted on the vertical axis, and their mean is plotted on the horizontal axis (dmean). The middle horizontal line represents the mean difference and the two horizontal lines, one above and the other below, are the 95% limits of agreement between measurements.

**Figure 2:**
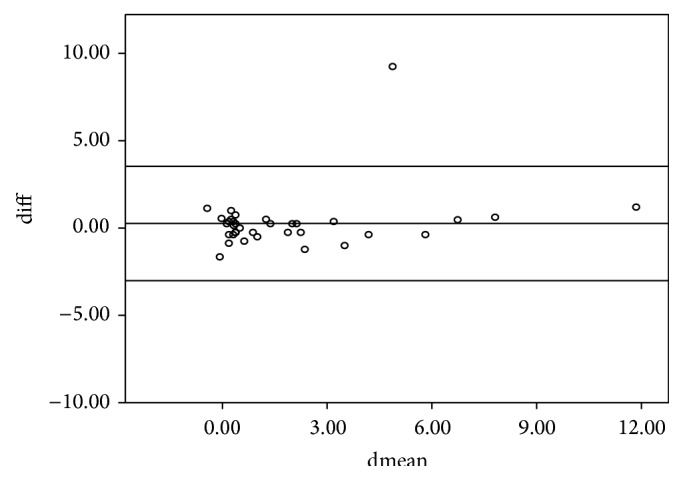
Bland-Altman plots comparing refractive measurements between autorefraction and subjective refraction (spherical equivalent).

**Figure 3:**
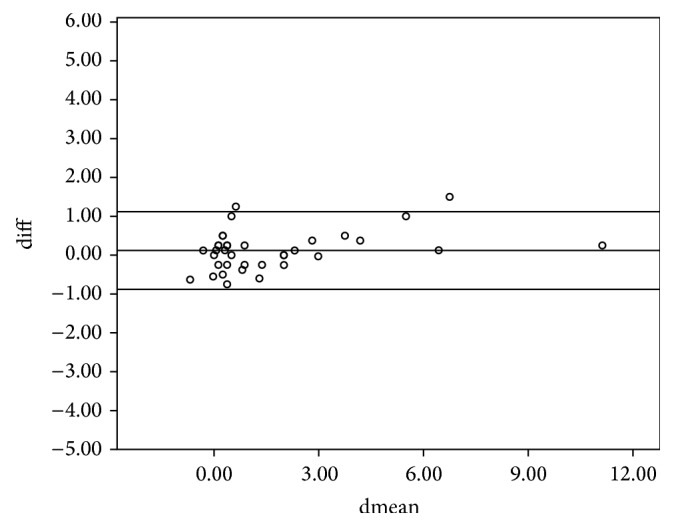
Bland-Altman plots comparing refractive measurements between retinoscopy and subjective refraction (spherical equivalent).

**Figure 4:**
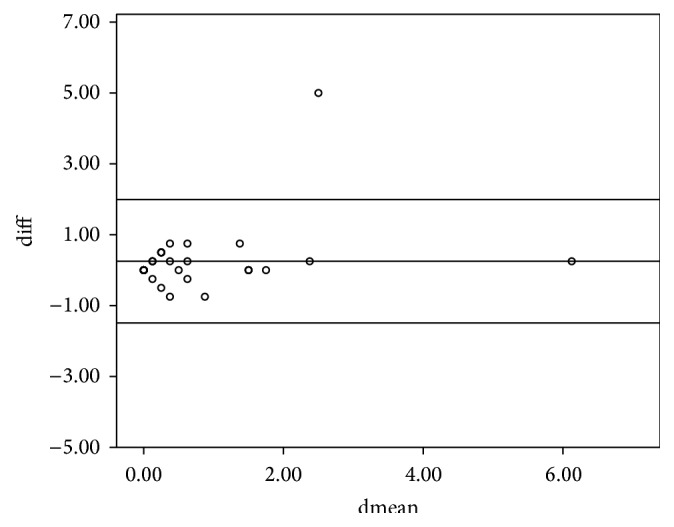
Bland-Altman plots comparing refractive measurements between retinoscopy and subjective refraction (total astigmatism).

**Figure 5:**
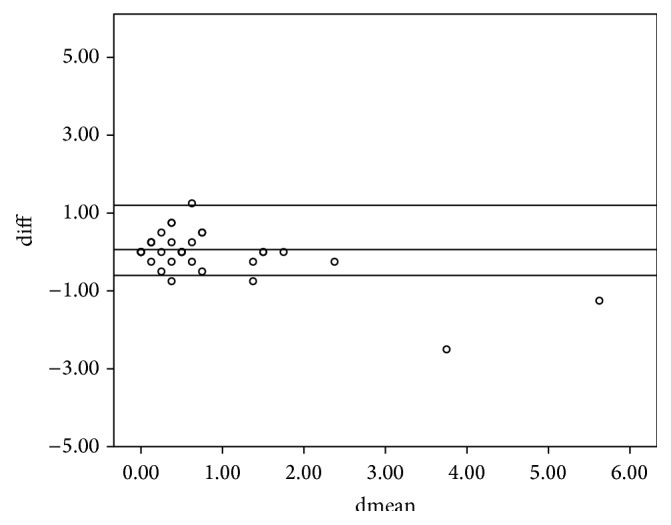
Bland-Altman plots comparing refractive measurements between autorefraction and retinoscopy (total astigmatism).

The study included 36 subjects of whom 14 were males and 22 were females. The mean age (SD) for the entire sample was 28 ± 16.5 years. The intraclass correlation coefficients (ICCs) of the various parameters between the left and right eyes are shown in Table 1. All ICCs were greater than 0.75 and hence the right eye was used in the analysis. The 95% limits of agreement and the intraclass correlation coefficient for the spherical equivalent between the various techniques are shown in Table 2. The intraclass correlation coefficients for the spherical equivalent between subjective refraction, autorefraction, and retinoscopy were 0.895 and 0.989, respectively. The mean differences between retinoscopy, autorefraction, and subjective refraction were statistically insignificant (*p* > 0.05) for the spherical equivalent. The Bland-Altman 95% limits of agreement between the subjective refraction and autorefraction; subjective refraction and retinoscopy; and retinoscopy and autorefraction for the spherical equivalent were −2.84 to 3.58, −0.88 to 1.12, and −3.01 to 3.53, respectively. Table 2 presents the intraclass correlation coefficient, mean difference, level of statistical significance, and the limits of agreement within each pair of techniques being compared for the spherical equivalent. All comparisons between techniques showed a mean difference with *p* > 0.05 which are represented graphically in Figures 1, 2, and 3. The intraclass correlation coefficients between spectacle total astigmatism and the following techniques were as follows: retinoscopy (0.85); autorefraction (0.92); Javal's rule (0.82); and Grosvenor et al. version (0.85). The Bland-Altman 95% limits of agreement between subjective refraction and autorefraction; subjective refraction and retinoscopy; subjective refraction and Javal's rule; and subjective refraction and Grosvenor et al. version were −0.87 to 1.25, −1.49 to 1.99, −0.73 to 1.93, and −0.89 to 1.7, respectively, for total astigmatism. The 95% limits of agreement and the intraclass correlation coefficient for total astigmatism between various techniques are shown in Table 3. Only the mean difference between “retinoscopy and subjective refraction” and “retinoscopy and autorefraction” had *p* > 0.05 for the total astigmatism. This is represented graphically in Figures 4 and 5. The mean differences between subjective refraction and autorefraction, and Javal's rule and Grosvenor et al. modification were statistically significant (*p* < 0.05). This is presented in Table 3. The agreement between cylinder axis of retinoscopy and subjective refraction was *K* = 0.64, *p* = 0.001 and between autorefraction and subjective refraction was *K* = 0.051, *p* = 0.38. This is shown in Table 4.

## 4. Discussion

In this study, Humphrey Zeiss 599 autorefractor/keratometer was utilized in obtaining the autorefraction and keratometry readings. This particular instrument has been utilized in several clinical studies and also shown to provide accurate and reliable readings [13, 14]. Its test retest reliability (ICC) exceeds 0.95 which makes it a suitable instrument for this study [13].

There was agreement between subjective refraction, retinoscopy, and autorefraction for the spherical equivalent as the mean differences between subjective refraction and either technique were not statistically different from zero. This implies there was agreement between the techniques for the spherical equivalent as further evidenced by the high intraclass coefficient between techniques. The ICC values are traditionally interpreted as follows: ICC of 0.4 indicates poor agreement; ICC from 0.4 to 0.75 indicates fair to good agreement; and ICC greater than 0.75 indicates good to exceptional agreement [16, 17]. Also, lower 95% limits of agreement indicate stronger agreement, provided the one-sample *t*-test was not significant for the mean difference between techniques being compared. From Table 2, it can be seen that retinoscopy had lower 95% limits of agreement with subjective refraction than autorefraction for the spherical equivalent. This implies retinoscopy agreed more with subjective refraction than autorefraction for the spherical equivalent. Also, the results indicate satisfactory agreement between autorefraction and retinoscopy in terms of the spherical equivalent.

There was also agreement between retinoscopy and autorefraction for the total astigmatism as evidenced by the statistically insignificant mean difference between the techniques and the high ICC between techniques. This indicates that autorefraction might be a suitable substitute objective refractive technique for a starting point in subjective refraction.

For estimating, total astigmatism no technique had a mean difference that was statistically insignificant except retinoscopy. This indicates only retinoscopy produced total astigmatism that substantially agreed with subjective refraction total astigmatism. All other techniques including autorefraction, Javal's rule, and Grosvenor modification did not exceptionally agree with subjective refraction in terms of the total astigmatism. Notwithstanding, all other techniques had ICC greater than 0.75 indicating some fair agreement with subjective refraction. Hence, they may be suitable alternative techniques in estimating the crude magnitude of astigmatism as a starting point in subjective refraction.

Autorefraction seems to have a higher intraclass correlation coefficient with subjective refraction than retinoscopy in estimating total astigmatism. Notwithstanding, the mean differences between total astigmatism on subjective refraction and retinoscopy were not statistically different from zero but those of autorefraction and subjective refraction were statistically different from zero. Though autorefraction had a lower mean difference than retinoscopy, the one-sample *t*-test for the autorefraction was significant because the standard error of mean for the autorefraction was smaller compared to retinoscopy.

The current study findings are consistent with those of previous studies that found agreement between autorefraction and retinoscopy [18–23]. This study showed that even though autorefraction predicted the magnitude of refraction well, it was not particularly good in estimating the axis of the cylinder. However, retinoscopy had excellent agreement with subjective refraction cylinder axis.

One of the limitations of this study is the relatively small sample size utilized in the study. Notwithstanding, the sample size utilized in this study can be considered adequate since several studies [24–27] of comparison between refraction techniques utilized smaller sample sizes compared to that of the current study.

In summary, the study showed moderate agreement between Javal's rule and subjective refraction total astigmatism. Autorefraction may be an appropriate starting point for subjective refraction but only retinoscopy may satisfactorily replace subjective refraction when subjective refraction is not applicable.

## Figures and Tables

**Table 1:** Intraclass correlation between right and left eyes for various parameters.

Parameter	Intraclass correlation coefficient	Confidence interval	*p*-value
Autorefraction spherical equivalent	0.934	0.871–0.966	*p* < 0.001
Autorefraction total astigmatism	0.921	0.845–9.960	*p* < 0.001
Retinoscopy spherical equivalent	0.929	0.861–0.964	*p* < 0.001
Retinoscopy total astigmatism	0.962	0.926–0.984	*p* < 0.001
Subjective refraction spherical equivalent	0.968	0.937–0.977	*p* < 0.001
Subjective refraction total astigmatism	0.952	0.913–0.977	*p* < 0.001
Total astigmatism by Javal's rule	0.783	0.562–0.892	*p* < 0.001
Total astigmatism by Grosvenor's version of Javal's rule	0.783	0.562–0.892	*p* < 0.001

**Table 2:** The Bland-Altman 95% limits agreement and intraclass correlation coefficient for spherical equivalent.

Parameter	Mean difference (standard deviation)	Bland-Altman 95% limits of agreement	*p* value	ICC (95% confidence interval)
Autorefraction versus retinoscopy	0.26 (1.67)	−3.01 to 3.53	0.348	0.902 (0.809–0.950)
Subjective versus retinoscopy	0.12 (0.51)	−0.88 to 1.12	0.157	0.989 (0.978–0.994)
Subjective versus autorefraction	0.37 (1.64)	−2.84 to 3.58	0.167	0.895 (0.796–0.947)

ICC = intraclass correlation coefficient.

**Table 3:** The Bland-Altman 95% limits agreement and intraclass correlation coefficient for total astigmatism.

Parameter	Mean difference (standard deviation)	Bland-Altman 95% limits of agreement	*p* value	ICC 95% confidence interval
Autorefraction versus subjective	0.19 (0.54)	−0.87 to 1.25	0.039	0.92 (0.84–0.96)
Retinoscopy versus subjective	0.25 (0.89)	−1.49 to 1.99	0.1	0.85 (0.699–0.921)
Javal's rule versus subjective	0.6 (0.68)	−0.73 to 1.93	<0.001	0.82 (0.23–0.93)
Grosvenor's modification versus subjective	0.4 (0.66)	−0.89 to 1.7	0.01	0.85 (0.6–0.933)
Autorefraction versus retinoscopy	0.06 (0.62)	−0.6 to 1.2	0.5	0.92 (0.857–0.963)

**Table 4:** Agreement between cylinder axis of subjective refraction and that of retinoscopy or autorefraction.

Parameter	Kappa	*p* value
Autorefraction versus subjective (exact)	0.051	0.38
Autorefraction versus subjective (within 5 degrees)	0.107	0.26
Retinoscopy versus subjective (exact)	0.64	<0.001
Retinoscopy versus subjective (within 5 degrees)	0.64	<0.001

## References

[1] Farook M., Venkatramani J., Gazzard G., Cheng A., Tan D., Saw S. M. (2005). Comparisons of the handheld autorefractor, table-mounted autorefractor, and subjective refraction in Singapore adults. *Optometry and Vision Science*.

[2] Salchow D. J., Zirm M. E., Stieldorf C., Parisi A. (1999). Comparison of objective and subjective refraction before and after laser in situ keratomileusis. *Journal of Cataract and Refractive Surgery*.

[3] Bullimore M. A., Fusaro R. E., Adams C. W. (1998). The repeatability of automated and clinician refraction. *Optometry and Vision Science*.

[4] Zhang M., Zhang R., He M. (2011). Self correction of refractive error among young people in rural China: results of cross sectional investigation. *British Medical Journal*.

[5] He M., Congdon N., MacKenzie G., Zeng Y., Silver J. D., Ellwein L. (2011). The child self-refraction study: results from urban Chinese children in Guangzhou. *Ophthalmology*.

[6] Jorge J., Queirós A., Almeida J. B., Parafita M. A. (2005). Retinoscopy/autorefraction: which is the best starting point for a noncycloplegic refraction?. *Optometry and Vision Science*.

[7] Choong Y.-F., Chen A.-H., Goh P.-P. (2006). A comparison of autorefraction and subjective refraction with and without cycloplegia in primary school children. *American Journal of Ophthalmology*.

[8] Prabakaran S., Dirani M., Chia A. (2009). Cycloplegic refraction in preschool children: comparisons between the hand-held autorefractor, table-mounted autorefractor and retinoscopy. *Ophthalmic and Physiological Optics*.

[9] Elliott M., Callender M. G., Elliott D. B. (1994). Accuracy of javal’s rule in the determination of spectacle astigmatism. *Optometry and Vision Science*.

[10] Grosvenor T., Quintero S., Perrigin D. M. (1988). Predicting refractive astigmatism: a suggested simplification of Javal's rule. *American Journal of Optometry and Physiological Optics*.

[11] Davies L. N., Mallen E. A. H., Wolffsohn J. S., Gilmartin B. (2003). Clinical evaluation of the Shin-Nippon NVision-K 5001/Grand Seiko WR-5100K autorefractor. *Optometry and Vision Science*.

[12] Cleary G., Spalton D. J., Patel P. M., Lin P.-F., Marshall J. (2009). Diagnostic accuracy and variability of autorefraction by the tracey visual function analyzer and the shin-nippon N Vision-K 5001 in relation to subjective refraction. *Ophthalmic and Physiological Optics*.

[13] Isenberg S. J., Signore M. D., Madani-Becker G. (2001). Use of the HARK autorefractor in children. *American Journal of Ophthalmology*.

[14] Cooper J., Citek K., Feldman J. M. (2011). Comparison of refractive error measurements in adults with Z-View aberrometer, Humphrey autorefractor, and subjective refraction. *Optometry*.

[15] Armstrong R. A. (2013). Statistical guidelines for the analysis of data obtained from one or both eyes. *Ophthalmic and Physiological Optics*.

[16] Rouse M. W., Borsting E., Deland P. N. (2002). Reliability of binocular vision measurements used in the classification of convergence insufficiency. *Optometry and Vision Science*.

[17] Fleiss J. L. (1986). *The Design and Analysis of Clinical Experiments*.

[18] Allen P. M., Radhakrishnan H., O'Leary D. J. (2003). Repeatability and validity of the PowerRefractor and the Nidek AR600-A in an adult population with healthy eyes. *Optometry and Vision Science*.

[19] Du Toit R., Soong K., Brian G., Ramke J. (2006). Quantification of refractive error: comparison of autorefractor and focometer. *Optometry and Vision Science*.

[20] Smith K., Weissberg E., Travison T. G. (2010). Alternative methods of refraction: a comparison of three techniques. *Optometry and Vision Science*.

[21] Davies L. N., Mallen E. A. H., Wolffsohn J. S., Gilmartin B. (2003). Clinical evaluation of the Shin-Nippon NVision-K 5001/Grand Seiko WR-5100 K autorefractor. *Optometry and Vision Science*.

[22] Sheppard A. L., Davies L. N. (2010). Clinical evaluation of the Grand Seiko Auto Ref/Keratometer WAM-5500. *Ophthalmic and Physiological Optics*.

[23] Lowery J. P., Joachim A., Olson R., Peel J., Pearce N. N. (2005). Autorefraction vs. retinoscopy: a comparison of non-cycloplegic measures in a pediatric sample. *Journal of Behavioral Optometry*.

[24] Twelker J. D., Mutti D. O. (2001). Retinoscopy in infants using a near noncycloplegic technique, cycloplegia with tropicamide 1%, and cycloplegia with cyclopentolate 1%. *Optometry and Vision Science*.

[25] Hopkins S., Sampson G. P., Hendicott P., Lacherez P., Wood J. M. (2012). Refraction in children: a comparison of two methods of accommodation control. *Optometry and Vision Science*.

[26] Steele G., Ireland D., Block S. (2003). Cycloplegic autorefraction results in pre-school children using the Nikon Retinomax Plus and the Welch Allyn Suresight. *Optometry and Vision Science*.

[27] Vasudevan B., Ciuffreda K. J., Meehan K., Grk D., Cox M. (2016). Comparison of objective refraction in darkness to cycloplegic refraction: a pilot study. *Clinical and Experimental Optometry*.

